# Mechanical Behavior of Cells within a Cell-Based Model of Wheat Leaf Growth

**DOI:** 10.3389/fpls.2016.01878

**Published:** 2016-12-15

**Authors:** Ulyana Zubairova, Sergey Nikolaev, Aleksey Penenko, Nikolay Podkolodnyy, Sergey Golushko, Dmitry Afonnikov, Nikolay Kolchanov

**Affiliations:** ^1^Department of Systems Biology, Institute of Cytology and Genetics (ICG), Siberian Branch of Russian Academy of ScienceNovosibirsk, Russia; ^2^Laboratory of Analysis and Optimization of Non-Linear Systems, Institute of Computational Technologies (ICG), Siberian Branch of Russian Academy of ScienceNovosibirsk, Russia; ^3^Laboratory of Mathematical Modeling of Hydrodynamic Processes in the Environment, Institute of Computational Mathematics and Mathematical Geophysics (ICM & MG), Siberian Branch of Russian Academy of ScienceNovosibirsk, Russia; ^4^Chair of Mathematical Methods in Geophysics, Faculty of Mechanics and Mathematics, Novosibirsk State UniversityNovosibirsk, Russia; ^5^Laboratory of Mathematical Problems of Geophysics, Institute of Computational Mathematics and Mathematical Geophysics (ICM & MG), Siberian Branch of Russian Academy of ScienceNovosibirsk, Russia; ^6^Chair of Informatics Systems, Faculty of Information Technologies, Novosibirsk State UniversityNovosibirsk, Russia; ^7^Chair of Mathematical Modeling, Faculty of Mechanics and Mathematics, Novosibirsk State UniversityNovosibirsk, Russia; ^8^Chair of Informational Biology, Faculty of Natural Sciences, Novosibirsk State UniversityNovosibirsk, Russia

**Keywords:** wheat leaf epidermis, symplastic growth, cell mechanics, osmotic and turgor pressure, autonomous cell growth, cell-based models

## Abstract

Understanding the principles and mechanisms of cell growth coordination in plant tissue remains an outstanding challenge for modern developmental biology. Cell-based modeling is a widely used technique for studying the geometric and topological features of plant tissue morphology during growth. We developed a quasi-one-dimensional model of unidirectional growth of a tissue layer in a linear leaf blade that takes cell autonomous growth mode into account. The model allows for fitting of the visible cell length using the experimental cell length distribution along the longitudinal axis of a wheat leaf epidermis. Additionally, it describes changes in turgor and osmotic pressures for each cell in the growing tissue. Our numerical experiments show that the pressures in the cell change over the cell cycle, and in symplastically growing tissue, they vary from cell to cell and strongly depend on the leaf growing zone to which the cells belong. Therefore, we believe that the mechanical signals generated by pressures are important to consider in simulations of tissue growth as possible targets for molecular genetic regulators of individual cell growth.

## 1. Introduction

Three fundamental processes characterize the development of multicellular plants: growth, differentiation, and morphogenesis. These processes result in an increase in the size and number of cells, the appearance of the structural and functional differences between them, and the formation of functionally specialized organs in plants. Plant science has accumulated a considerable amount of information about plant growth and plant genetics. Nevertheless, the molecular genetic mechanisms that influence the growth and development of plants are still unclear. Analysis and reconstruction of the dynamics of genetic regulatory networks are only the first steps toward understanding how genetic information determines the morphogenesis of plants (Kalve et al., 2014; Chaiwanon et al., 2016). At present, increasingly more facts demonstrate that full understanding of the mechanisms of morphogenesis requires considering the processes that occur at the tissue level in addition to the information on the expression of genes. These processes are involved in spatial pattern formation (Swarup et al., 2005; Jönsson et al., 2006; Bayer et al., 2009) and result in strains and stresses in cell walls (Green, 1999; Hamant et al., 2008). Understanding the relationships between the processes at the molecular-genetic level and at the level of cell ensembles and tissues and how these relationships result in the implementation of the biological function and morphogenesis is perhaps the most central question in systems biology (Noble, 2008).

The ability of cells to stretch and to change shape is the basis for the functional specialization of tissue and the formation of structure in plants. In contrast to animal cells, plant cells cannot migrate during tissue growth. Therefore, these cells achieve perfect control over the final shape by modulating cell division and expansion (Cosgrove, 2005). Knowledge about regulation of the biomechanical properties of the cells during these processes is now widely used in studies of plant morphogenesis. The ability of plant cells to detect mechanical stresses in a tissue and to use them as control signals is considered to be the foundation of regulated growth and morphodynamics (Ali and Traas, 2016; Braybrook and Jönsson, 2016). It is believed that management strategies based on mechanosensing include both stabilization due to the negative feedback and an increase of differences in the rates and directions of neighboring cell growth due to positive feedback (Sassi and Traas, 2015). However, despite intensive studies of such regulation (Hamant and Traas, 2010; Robinson et al., 2013; Routier-Kierzkowska and Smith, 2013), the question about the mechanisms governing the growth of the cell biomass is still open.

Experimental studies have shown a tight relationship between biomechanics and molecular-genetic processes. Such works include studies on how mechanical signals affect the cell cycle (Streichan et al., 2014), how cellular differentiation depends on the different mechanical properties of substrates (Wells, 2008; Lv et al., 2015), and how the mechanical properties of cells are involved in cancer transformation (Lekka et al., 2012). Modern experimental techniques, particularly techniques based on atomic force microscopy (Milani et al., 2011, 2013; Sugimura et al., 2016), allow for measuring the mechanical properties of the cell wall and evaluating turgor pressure in certain plant cells, which is an important factor in the internal mechano-regulation of growth processes and cell wall extension (Ali and Traas, 2016). However, experimentally studying the dynamics of mechanical parameters in individual cells in growing plant tissue is a difficult task.

Computer modeling allows the accumulated knowledge about complex spatio-temporal interactions of biomechanical and morphogenetic processes that regulate the growth and functioning of cells, tissues, organs and body to be combined into a single conceptual scheme. Currently, cell-based models are widely used for simulating the growth of different plant organs and tissues. Examples include extensions of the cellular Potts model (Graner and Glazier, 1992; Glazier and Graner, 1993) and vertex-based model (Nagai and Honda, 2001; Merks et al., 2011; Shapiro et al., 2013). The dynamics of tissue morphology in these models is determined by the mechanics of motion of elements of cell borders (border pixels for Potts model and vertex coordinates of piecewise linear cell-cell borders for vertex-based models). In these examples, the mechanical behavior of cells is determined by setting the energy functions with respect to the “target” values of the cell volumes and boundaries. Changes of the “target” parameter may depend on time and/or state variables, which simulate cell growth. In the framework of the vertex-based models, one can describe geometric properties of the cellular structure of the tissue and the mechanical state of the cells in the tissue in terms of stretches and compressions of its volumes and boundaries. At the same time, it is impossible to explicitly express turgor and osmotic pressures in terms of the state variables of these models. Considering the importance of investigating the relationship between the mechanical properties of cells within tissues and its molecular-genetic characteristics, the development of mechanical approaches that take the internal state of the cell into account is promising for use in cell-based models of plant tissue morphodynamics.

In this paper, we propose a cell-based model for the growth of monocot leaf epidermis. The model is an extension of our previous work (Zubairova et al., 2015), which takes the difference between cell growth and division rates in different parts of the leaf growth zone into account. By taking the geometrical characteristics of tissue into account, we constructed the model state variables so that we could explicitly express turgor and osmotic pressures in the cells. This makes it possible to study the distribution of the pressures in cells of growing tissue, possible mechanisms of regulation of coordinated cellular growth, and other issues of mechanics of plant tissue growth. Using the growth of wheat leaf epidermis as an example, we showed that our model allows us to approximate the experimental cell length profile along the growth axis of the leaf (Beemster et al., 1996); at the same time, the relationship between the state variables of the model indicate significant fluctuations of pressure in the cells of the leaf growth zone. This opens up prospects for further research of the role of pressure and stress in growing cells as biomechanical regulators of molecular-genetic systems of cell morphogenesis.

The remainder of this paper is organized as follows. In Section 2 (Methods), we focus on the description of a mathematical model for the growth of wheat leaf epidermis, parameter estimation and implementation. In Section 3 (Results), we present the results of computational experiments on approximating the experimental cell length profile in a real wheat leaf and analyze the mechanical behavior of cells during symplastic growth. In Section 4 (Discussion), we discuss our simulation results with the conclusions of this work.

## 2. Methods

### 2.1. A mathematical model of symplastic unidirectional growth of a linear leaf

#### 2.1.1. Biomechanics of the autonomous growth of a single plant cell

At present, experimental facts and theories accumulated in the literature allow us to formulate the following idea about the mechanics of plant cell growth (Equations 1–6). The concentration, *c*, of the dry biomass, *m*, in the cell changes due to the biosynthesis (growth function *F*_*grow*_) and by varying the cell's volume, *V*:

(1) $$\begin{array}{l} \frac{dc}{dt}=F_{grow}+\frac{\partial c}{\partial V}\cdot \frac{dV}{dt} \end{array}$$

For simplicity, we assume that the cell biomass composition does not change qualitatively and that the concentration of osmolytes is proportional to the concentration of dry biomass. In particular, we can assume that this concentration is equal to *c*. In this case, the osmotic pressure inside the cell can be estimated from the Van't Hoff equation (Nobel, 2009):

(2) $$\begin{array}{l} P_{osm}^{in}=c\;R\;T, \end{array}$$

where *T* is the absolute temperature and *R* is the universal gas constant.

Osmotic pressure causes the inflow of water into the cell, which simultaneously stretches the elastic cell wall. This gives rise to mechanical stress in the wall and thus to hydrostatic (turgor) pressure inside the cell:

(3) $$\begin{array}{l} \sigma_{V}P_{turg}=\frac{V-Vr}{Vr}, \end{array}$$

where σ_*V*_ is the elastic flexibility of the cell chamber, *V* is the visible cell volume, and *Vr* is the relaxed volume of the cell chamber, i.e., the volume that will take the cell chamber bounded by the cell wall if the cell is placed into a hyperosmotic solution (in this case, the cell will lose turgor and the cell wall will cease to be in the stress-strain state).

The flow of water into the cell occurs when the difference between the osmotic pressures inside and outside the cell is greater than the turgor pressure:

$$\Psi_{w}=\left(P_{osm}^{in}-P_{osm}^{out}\right)-P_{turg}>0,$$

where −Ψ_*w*_ is the water potential of the cell relative to the environment (Nobel, 2009) and $P_{osm}^{out}$ is the osmotic pressure in the medium around the cell.

The change of the visible cell volume, *V*, is proportional to the water flow inside the cell:

(4) $$\begin{array}{l} \frac{dV}{dt}=S\;L_{w}\;\Psi_{w}, \end{array}$$

where *S* is the cell surface area through which the water enters the cell and *L*_*w*_ is the hydraulic conductivity of the cell wall (Nobel, 2009). According to Ortega (2010), the relative change of the cell chamber can be represented as the sum of the irreversible changes in the volume of the cell chamber (actual growth) and its reversible elastic deformation:

(5) $$\begin{array}{l} \frac{dV}{Vdt}=\phi \left(P_{turg}-P_{c}\right)+\sigma \frac{dP_{turg}}{dt}, \end{array}$$

where ϕ is the irreversible wall extensibility and *P*_*c*_ is the threshold turgor pressure.

In our model, instead of Equation (5), we introduced explicit expressions for the osmotic and turgor pressures (will be introduced below, Equations 7, 8) and postulated the following function for the cell wall growth rate. Specifically, with an increase in the turgor pressure above a certain threshold, *P*_*c*_ (which is different for different types of cells), the biosynthesis of the cell wall material begins (Dyson et al., 2012). This material is delivered into the wall, and it begins to grow with a rate determined by the function Φ, dependent on the turgor pressure exceeding a certain threshold, *P*_*c*_:

(6) $$\frac{dVr}{dt}=\{\begin{array}{cc} \;0, & \text{ if }P_{turg}\leq P_{c}\\ \Phi (P_{turg}-P_{c}), & \text{otherwise} \end{array}$$

A cell of linear leaf blade epidermis is represented in the model as a parallelepiped with a volume *V* = *l* · *r*_1_ · *r*_2_. To simplify the model, we assume that the cell thickness *r*_1_ and the cell width *r*_2_ do not change during the growth process and are equal to *r*. Therefore, we will consider a unidirectional growth of a plant cell. This allows us to describe the mechanics of cell growth in terms of lengths as follows: visible cell length, *l*; relaxed cell length (the cell wall length in the unstressed state), *lr*; and isosmotic cell length, *li*.

The isosmotic cell length, *li*, is defined as follows. As discussed above, the amount of osmolytes in the cell is equal to *m* = *c* · *V*. Suppose that the osmotic pressure of the cell's environment is $P_{osm}^{out}$ and that the concentration of osmolytes in the cell's environment is *c*^*out*^. For the cell to become isotonic to the environment, the cell volume should satisfy the equality *m* = *c*^*out*^ · *Vi*; hence, $c=c^{out}\cdot \frac{Vi}{V}$.

In this case, the difference between osmotic pressures in and out of the cell can be expressed as follows: $P_{osm}^{in}-P_{osm}^{out}=\left(c-c^{out}\right)\;R\;T=c^{out}\;R\;T\;\left(Vi-V\right)/V$. Substituting *V* = *l* · *r*^2^ and *Vi* = *li* · *r*^2^, we obtain

(7) $$\begin{array}{l} P_{osm}=P_{osm}^{in}-P_{osm}^{out}=\alpha \;\frac{li-l}{l}, \end{array}$$

where α = *c*^*out*^ *R T* is the coefficient of osmotic pressure. Note that by assuming a constant cell protoplast composition, we can write the variable *m* = *c*^*out*^*r*^2^ · *li*, i.e., cell dry biomass is proportional to isosmotic length, *li*.

In the case of unidirectional growth, the turgor pressure can be expressed as follows:

(8) $$\begin{array}{l} P_{turg}=\beta \;\frac{l-lr}{lr}, \end{array}$$

where $\beta =\frac{S_{w}}{S_{c}}\;E$ is the coefficient of turgor pressure. *S*_*w*_ is the cross-sectional area of the cell wall, and when the cell wall thickness, *d*_*w*_, is small enough, *S*_*w*_ = 4*r* · *d*_*w*_. $S_{c}=r^{2}$ is the cross-sectional area of the cell across its length, and *E* is the Young's modulus of the cell wall material.

Suppose that water flows into the cell through the lower facet surface *r* · *l*. Then, following Ortega (2010), express the growth rate of the visible cell length, *l*, by the following equation:

(9) $$\begin{array}{l} \frac{dl}{dt}=r\cdot l\cdot L_{w}\cdot \left(P_{osm}-P_{turg}\right), \end{array}$$

or for specific growth rate:

(10) $$\begin{array}{l} \frac{dl}{l\;dt}=r\cdot L_{w}\cdot \left(\alpha \;\frac{li-l}{l}-\beta \;\frac{l-lr}{lr}\right). \end{array}$$

Defining the function Φ from Equation (6), we assumed that the growth rate of the relaxed cell length depends on the growth rate of the cell biomass and is identified as:

(11) $$\frac{d\;lr}{dt}=\{\begin{array}{cc} \;0, & \text{ if }P_{turg}\leq P_{c}\\ \eta \;\frac{d\;li}{dt}\;{\left(P_{turg}-P_{c}\right)}^{3}, & \text{otherwise,} \end{array}$$

where η is the proportional coefficient between the cell wall and the biomass growth rates. The specific form of the regulatory function Φ is discussed in Section 4.3.

We consider that the increase in the isolated cell dry biomass, *m*, under some standard conditions is described by a fixed function of time, *m*(*t*) = *F*_*m*_(*t*), and we will refer to it as *autonomous growth* of isolated cells. Taking the assumptions of our model into account, we define the isosmotic cell length, *li*, as an explicit function of time:

(12) $$\begin{array}{l} li\left(t\right)=F_{grow}\left(t\right). \end{array}$$

For the model of the autonomous growth of a single plant cell, we used a linear function of time for the cell isosmotic length growth, *F*_*grow*_(*t*) = *a* (*t* − *t*_0_) + *b*, where *a* is the growth rate and *b* is the initial cell size. The choice of a linear growth function will be explained in more detail in the Section 4. Therefore, the model of the unidirectional autonomous growth of a single plant cell is defined by Equations (10–12).

#### 2.1.2. Mechanics of symplastic unidirectional growth of cells within the leaf epidermis

**Figure 1:**
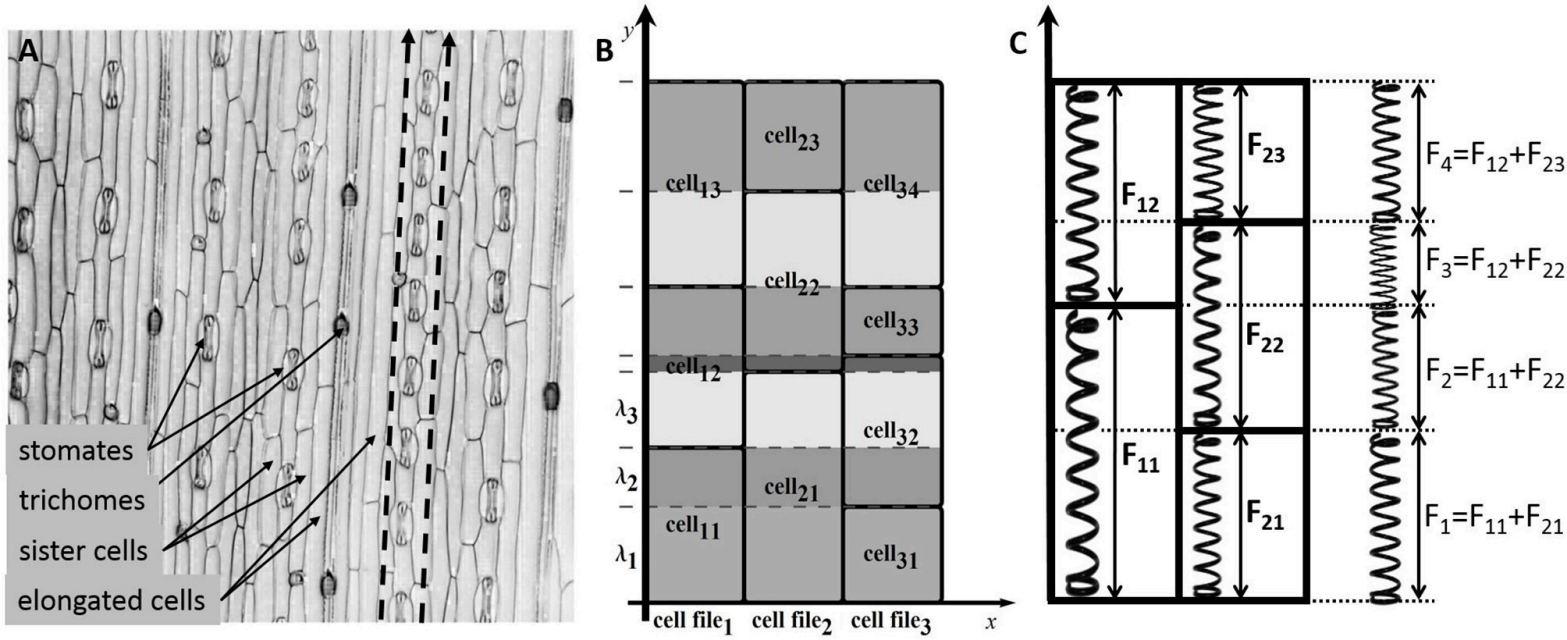
**The cell structure of the wheat leaf epidermis. (A)** Micro-photograph of the wheat leaf epidermis (constructed from a 3D confocal image of the wheat leaf epidermis, supplied courtesy of A. Doroshkov), where dashed arrows mark cell files. **(B)** The scheme of partitioning of cells of the “model leaf” into fragments {λ_1_, λ_2_, λ_3_, …}. With this partition, e.g., the fragment λ_2_ belongs to the cells *cell*_11_, *cell*_21_, and *cell*_32_. **(C)** The force stretching a fragment is the sum of forces stretching the cells that include the fragment.

In this paper, we studied plant tissue growth based on a simplified model of wheat leaf epidermis (Figure 1A) composed of cell files consisting of similar cells. We assumed that the cells within the leaf epidermis grow in optimal conditions, its growth is described by the same time-dependent function of growth for isosmotic length as for an isolated cell, and it has the same mechanical parameters (Table 1). The only additional condition is that its walls are “glued” with walls of neighboring cells. Since neighboring cells can grow at different rates, common fragments of its walls cause additional mutual stresses within each other. Consequently, the growth rate of a cell wall fragment is actualized as being different from the free growth rates of respective cells, and hence, the growth of the cell wall can be nonuniform within the cell.

**Table 1:** **Mechanical parameters of the model**.

**Parameter**	**Description**	**Value**	**Sensitivity**	**References**
*L*_*w*_	Hydraulic conductivity of the cell wall	40 μm^−1^ · h^−1^ · bar^−1^	0.00146894	Nobel, 2009
α	Coefficient for osmotic pressure	10 bar	5.36703	Nobel, 2009
β	Coefficient for turgor pressure	100 bar	−0.000028689	Nobel, 2009; Gibson, 2012
η	Coefficient of cell wall growth rate	0.15	53.9229	Gibson, 2012
*P*_*c*_	Threshold of turgor pressure	2 bar	−14.5014	Nobel, 2009

*The symbols of the parameters are listed in the first column, a parameter description in the second column, the selected parameter value in the third column, the sensitivity of visible cell length to the parameter in the fourth column, and literature reference in the last column*.

In the model, leaf epidermis is represented as a “brickwork” (Figure 1B) consisting of brick-like cells of the same type arranged in longitudinal cell files. Within each cell file, all cells have the same constant cross section and different lengths (due to different growth rates of cells), and the “model leaf” has a rectangular shape in plan. Because of such a simple topology, despite the fact that we are interested in the surface of the leaf, we can consider the tissue in the model not as a two-dimensional object but as a set of one-dimensional cell chains, which are glued together. The last fact accounts for the symplastic mode of growth.

To formalize the geometric model of linear leaf epidermis, consider a rectangular compartment of rectangular cells (Figure 1B) in 0*xy* coordinates. Assume that the cells grow in the 0*y* direction. Let us mark cell files in the 0*x* direction with index *n* (*n* = 1, …, *N*), mark the cells in each file in the 0*y* direction with index *m* (*m* = 1, …, *M*_*n*_), and designate the length of the *m*-th cell in the *n*-th file as *l*_*mn*_. We divide each cell into fragments to take the symplastic mode of growth into account in the model. The procedure is as follows: we extend the “horizontal” border of each cell parallel to the 0*x* axis throughout the leaf blade such that the leaf is divided into fragments in the 0*y* direction. Index the fragments with index *k* (*k* = 1, …, *K*) and denote the *k*-th fragment length as λ_*k*_.

From a mechanical perspective, the fragment λ_*k*_ is glued fragments of different cells undergoing a force determined by the turgor pressure in the corresponding cells. Hence, the entire fragment experiences the results of all these forces (Figure 1C). To simplify the model, we assume that the parameters determining the mechanical behavior of the model (*L*_*w*_, η, α, β) have the same values for all cells of the leaf. In this case, the next formula defines the growth rate of the common fragment for all cells contained in it.

(13) $$\begin{array}{l} \frac{d\lambda_{k}}{dt}=\frac{\lambda_{k}}{N}\overset{N}{\underset{n=1}{\sum }}{\left(\frac{dl_{mn}}{l_{mn}dt}\right)}_{free},\;\left(m:\;\lambda_{k}\in l_{mn}\right), \end{array}$$

where *l*_*mn*_ is the visible length of cell *m* in cell file *n* and the expression ${\left(\frac{dl_{mn}}{l_{mn}dt}\right)}_{free}$ denotes the specific growth rate of the cell visible length at the given moment if the cell would not have mechanical bonds with neighboring cells. This rate is determined by Equation (10) for the corresponding cell *m* in the cell file *n*.

The visible length of the cell, *l*_*mn*_, within the leaf epidermis is the sum of lengths of fragments, λ_*k*_, belonging to it; thus, its derivative is also equal to the sum of derivatives of fragment lengths:

(14) $$\begin{array}{l} \frac{dl_{mn}}{dt}=\underset{\lambda_{k}\in l_{mn}}{\sum }\frac{d\lambda_{k}}{dt}=\underset{\lambda_{k}\in l_{mn}}{\sum }\frac{\lambda_{k}}{N}\overset{N}{\underset{n=1}{\sum }}{\left(\frac{dl_{mn}}{l_{mn}dt}\right)}_{free} \end{array}$$

In our model, we assume autonomous growth of the cell within the leaf epidermis; therefore, the isosmotic cell length, *li*_*mn*_, and the relaxed cell length, *lr*_*mn*_, are regulated by each cell in the same way as for an isolated cell. The growth of the isosmotic cell length, *li*_*mn*_, is defined by an explicit function of time and by the following initial data: the moment of time, *t*_0_, when the cell appeared with its initial isosmotic length, $li_{mn}^{0}$.

#### 2.1.3. Longitudinal zonation pattern of the leaf growth zone

Longitudinal growth of wheat leaf occurs in a growth zone, which is a relatively short part of the leaf located at its base. The growth zone consists of a division zone (DZ), or meristem, and elongation zone (EZ) (Beemster et al., 1996). Note that the length of the meristem (DZ) is significantly smaller than the length of the elongation zone. Due to permanent division of cells in the DZ, the average length of a cell in the DZ is almost identical in space and time. With the transition into the EZ, cell proliferation stops and cells begin to stretch rapidly in the longitudinal direction. At the end of the EZ, the stretching stops. The described cell behavior is supported by the experimental measurements of cell lengths in wheat leaf (Beemster et al., 1996; Tardieu et al., 2000; Hu and Schmidhalter, 2008).

In our model of the linear leaf blade growth, we assume a stationary longitudinal zonation pattern of the leaf: a division zone (DZ) and an elongation zone (EZ). Our assumption of the stationary location of the two zones with respect to the leaf base is based on the model of switching between two zones due to morphogenetic mechanisms. For instance, such a model was used in the work of Vos et al. (2014) in the simulation of *Arabidopsis thaliana* root growth. Similar molecular mechanisms were used for justifying the stable location of the growth zone size in the shoot apical meristem of *A. thaliana* in our earlier work (Nikolaev et al., 2011). Here, we assumed that there exists some morphogen synthesized at the leaf base and propagated to the leaf tip with the constant gradient concentration providing the stable zonation pattern during leaf growth.

#### 2.1.4. Division of cells in the division zone

The cell in the division zone divides when its isosmotic length, *li*_*mn*_, reaches a critical value, *li*_*max*_. The division of the cell is described by rewriting of the cell parameters in the following way: (1) isosmotic, visible, and relaxed lengths of the mother cell are distributed between two daughter cells with the proportion of *d*/(1−*d*), where *d* is the division factor; (2) the initial isosmotic lengths, *li*_0_, of daughter cells are *d* · *li*_*max*_ and (1 − *d*) *li*_*max*_; and (3) the birth time, *t*0, of two daughter cells is set to *t*, the time of mother cell division. The division factor, *d*, is a random variable with the truncated normal distribution from the interval (0.1, 0.9) with mean μ_*d*_ = 0.5 and standard deviation σ_*d*_ = 0.1. These parameters for the division factor distribution are typical for the simulations of plant tissue growth (see, for example, Chickarmane et al., 2012).

### 2.2. Parameter estimation and sensitivity analysis

#### 2.2.1. Mechanical parameters

All the parameters determining the mechanics of the cell were obtained using literature data and adopted to provide a cell cycle duration of 24.7 h (Beemster et al., 1996). The value of the hydraulic conductivity of the cell wall, *L*_*w*_, was set to 40μm · h^−1^ · bar^−1^. This estimate is within the range proposed by Nobel (2009) for *L*_*w*_ (10^−13^ to 2 · 10^−12^ m · s^−1^ · Pa^−1^, i.e., 36–720 μm · h^−1^ · bar^−1^ in the units used here). Other authors suggest similar values for this parameter (Hukin et al., 2002; Mishra, 2004).

To estimate the coefficient for osmotic pressure from Equation (7), α, we used equation α = *c*^*out*^ *R T*, where *R* = 8.31446 J mol^−1^ K^−1^ is the gas constant and *T* = 300 K is the temperature in Kelvin. We used *c*^*out*^ = 0.25 M, which is close to the estimate of cell sap osmolarity as 0.3 M (Nobel, 2009), yielding α = 10 bar.

Estimates of the cell wall Young's modulus, *E*, from the literature vary from ≈ 1 to 10, 000 bar (Krupinski et al., 2016); in our simulations, we used *E* = 1000 bar. To estimate the coefficient for turgor pressure from Equation (8), β, we used equation $\beta =\frac{S_{w}}{S_{c}}\;E=\frac{4r\cdot d_{w}}{r^{2}}\;E$, assuming *r* = 4μm and *d*_*w*_ = 0.1 μm. This yields β = 100 bar.

The threshold of turgor pressure when the cell begins to grow, *P*_*c*_ = 2 bar, is close to the values widely discussed in the literature as the “wall yield threshold” (Nobel, 2009; Dyson et al., 2012).

We have not found any estimates of the coefficient of the cell wall growth parameter, η, in wheat leaves in the literature. Therefore, we conducted a numerical experiment to obtain its estimate with a single cell model (see Equations 10–12). We changed the value of η in the range of (0, 1) and estimated the turgor and osmotic pressures in the cell during the cell cycle. The value of η = 0.15 yielded the minimal absolute deviation of these two pressures and was chosen as the reasonable η estimate. We summarize the parameter values in Table 1.

To estimate the sensitivity of the cell visible length with respect to the mechanical parameters of the model, we simulated single cell growth during 24 h using Equations (10–12) with varied parameters at each run. The changes of parameters were within 10% of their estimated values with a constant step. The sensitivity was estimated as the ratio of the visible length deviation to the deviation of the parameter value.

#### 2.2.2. Kinetic parameters

The model parameters defining the cell growth rate were obtained using experimental data on the cell length profile in a wheat leaf from Beemster et al. (1996). Beemster et al. (1996) reported the lengths of all cells along a file of sister cells (file adjacent to stomatal cell files) within wheat leaf epidermis. In their work, data were presented as follows: the individual lengths of successive cells were averaged over 0.5, 1.0, and 2.0 mm *intervals* in the basal part of the leaf; the next more distal; and the remaining, most distal part of the growth zone, respectively. Data on sizes of leaf growth zones, average cell lengths in each zone, and average cell cycle for one of the leaves are summarized in Table 2.

**Table 2:** **Experimental data on the growth of the wheat leaf from the work of Beemster et al. (1996), which were used for obtaining the model parameters**.

**Parameter**	**Value**
The average length of the growth zone (GZ), mm	23.8
The average length of the division zone (DZ), mm	3.3
The average length of the elongation zone (EZ), mm	20.5
${\hat{t}}_{div}$, the average cell cycle duration (for a cell from the division zone), hour	24.7
${\hat{t}}_{elong}$, the average elongation period (for a cell from the elongation zone), hour	71.09
${\hat{l}}_{0}$, the visible average initial cell length in the division zone, μm	14.74
${\hat{l}}_{div}$, the visible average final cell length in the division zone, μm	29.48
${\hat{l}}_{elong}$, the visible average final cell length in the elongation zone, μm	197.2

According to our model, all cells have uniform growth rates, and individual initial sizes determine different cell cycle durations. We suppose that a cell changes its growth rate once it reaches the critical value, *li*_*max*_, and it results in a piecewise-linear function for isosmotic length changes during cell growth (**Figure 3** inset):

(15) $$\begin{array}{ccc} li_{mn}(t) & \text{​}=\text{​} & \{\begin{array}{cc} a_{1}(t-t_{0_{mn}})+li_{mn}(t_{0_{mn}}), & \text{if }t_{0_{mn}}\leq t<t_{0_{mn}}\\ \; & +t_{div_{mn}}\\ a_{2}(t-t_{0_{mn}}-t_{div_{mn}})+li_{max}, & \text{if }t_{0_{mn}}+t_{div_{mn}}\leq t<\\ \; & t_{0_{mn}}+t_{div_{mn}}+t_{elong_{mn}} \end{array} \end{array}$$

where the coefficients *a*_1_ and *a*_2_ are the growth rates of the cells in DZ and EZ, respectively; *t*_0_*mn*__ is the time point when the cell appeared; *t*_0_*mn*__ + *t*_*div*_*mn*__ is the time point when the cell isosmotic length achieves the critical value; and *t*_0_*mn*__ + *t*_*div*_*mn*__ + *t*_*elong*_*mn*__ is the time point when the cell exited the EZ.

The critical point of the estimation is next. The experimental data are the distribution of the average *visible* cell lengths along the cell file (see Table 2), whereas we need to estimate the dynamics of the “*internal”* variable, the isosmotic length growth function. The experimental data provide values of the average cell cycle duration, ${\hat{t}}_{div}$; the average elongation period, ${\hat{t}}_{elong}$; and values of average visible length at these moments.

The coefficients *a*_1_ and *a*_2_ were obtained numerically. They are chosen to obtain the best fit between the cell length distributions in the model and real wheat leaf epidermis (Beemster et al., 1996). The cost function is based on the squared difference of the average length of the cells in the model, ${\bar{l}}_{i_{model}}$, and real leaf, ${\bar{l}}_{i_{exp}}$, within the *i*th interval of distance from the leaf base weighted with the scaling factor *k*_*i*_:

(16) $$\begin{array}{l} F_{cost}=\underset{i\in intervals}{\sum }k_{i}\cdot {\left({\bar{l}}_{i_{model}}-{\bar{l}}_{i_{exp}}\right)}^{2}. \end{array}$$

where the scaling factors, *k*_*i*_, are proportional to the interval number and provide greater weight to the intervals for greater distances from the base. This weighting is introduced to balance the cost contribution from the large number of small cells in the DZ and small number of large cells at the distal part of the EZ.

The search for the *a*_1_ and *a*_2_ estimates starts from the initial values. These values were obtained by numerically solving Equations (10–12) using Mathematica software to obtain the solutions of the inverse problem for the model of a single cell growth, providing that the visible cell length at the time moments ${\hat{t}}_{div}$ and ${\hat{t}}_{elong}$ are equal to its experimental estimate of ${\hat{l}}_{div}$ and ${\hat{l}}_{elong}$ (Table 2). Next, we simulated the epidermis growth until its length becomes equal to that of the wheat leaf (Table 2). The iterative search of the minimum of the cost function Equation (16) was performed numerically by the grid enumeration of the *a*_1_ parameter with subsequent adjustment of the *a*_2_ using the golden ratio method under fixed *a*_1_. Consequently, we obtained *a*_1_ = 0.69μ m · h^−1^ and *a*_2_ = 5.04 μm · h^−1^.

### 2.3. Model implementation

The computational model for the growth of wheat leaf epidermis was developed using a modified formalism of differential L-systems (DL-systems) (Lindenmayer, 1968; Prusinkiewicz et al., 1993), which we termed “glued” DL-systems. Implementation details are provided in Zubairova et al. (2015).

Briefly, the model consists of two types of DL-systems: cellular and fragmental. The first type describes the growth and division of the cells and is represented by several 1D DL-systems. Each 1D DL-system corresponds to the cell file of the 2D linear leaf blade (Figure 1B). *N* cellular DL-systems provide the cellular structure of a linear leaf blade consisting of *N* parallel files that cannot slide with respect to its neighbors (i.e., “glued”). The alphabet of the cellular DL-system consists of tree symbols corresponding to the cells from three zones: DZ, TZ, and EZ. Each symbol is supported by the following parameters: isosmotic length, *li*; visible length, *l*; relaxed length, *lr*; initial isosmotic length, *li*_0_; and initial time, *t*_0_.

The alphabet of the fragmental DL-system consists of one symbol, the cell fragment (Figure 1B). Its parameters are the fragment length, λ, and the matching vector of the fragment and cells numbers in each cell file sharing this fragment. The dynamics of the parameters *li*, *l*, *lr*, and λ for the corresponding cells and fragments are determined by Equations (11–14). The rewriting rules are common for cellular and fragmental DL-systems, and they come into action at the moment of division of any cell from the DZ. Thus, an additional fragmental DL-system coordinates the operation of cellular DL-systems supporting the symplastic growth of the leaf blade.

This formalism of the “glued” DL-systems results in a quasi-one-dimensional representation of the leaf blade tissue and allows one to simulate efficiently the growth of the real size leaves (see Table 2). It can handle the cell numbers comparable with that of the real leaf.

The computational model was implemented in an in-house-developed open source software including the implementation of algorithms in C ++ and Wolfram languages (Mathematica). The source code is available upon request.

### 2.4. Statistical data analysis

Descriptive statistical analysis and data visualization were performed using Mathematica 10 package. Additionally, we performed cluster analysis of the pressure profiles for the cells during the leaf growth simulation. The pressure profile is a vector of pressure values recorded for the cell from its birth until its division or until simulation stops. The size of the vector is equal to 150, which is double the maximal observed cell lifespan (75 h) during the simulation. A single element corresponds to a 1 h time interval. The first 75 numbers in the vector represent osmotic pressure values; the others represent the turgor pressure values. All the vector elements corresponding to time steps after cell division were set to 0. To determine groups of cells with similar pressure profiles, we used k-mean clustering using the Clustering Components function from the Mathematica 10 package. Clustering was applied to the set of pressure profiles using a distance function based on the number of point changes needed to go from one curve to another. The number of clusters was set to 10.

## 3. Results

### 3.1. The parameters of the model and its robustness

**Figure 2:**
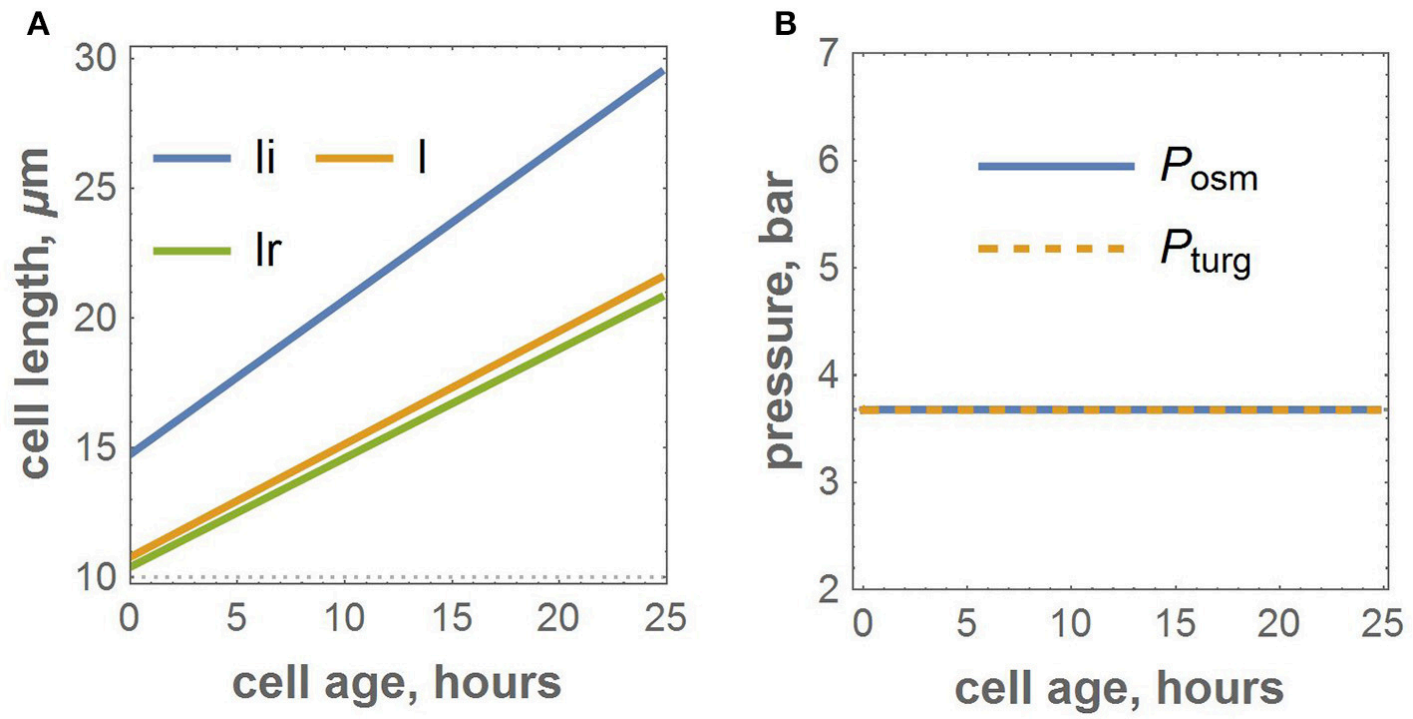
**Numerical experiment for the growth of the isolated cell with the adopted parameters. (A)** Growth functions for isosmotic, visible, and relaxed lengths. **(B)** The dynamics of osmotic and turgor pressures.

We proposed a cell-based model for the growth of linear leaf epidermis, which is based on the explicit expression of turgor and osmotic pressures during cell growth. The model depends on the number of parameters that describe the mechanical and kinetic properties of the cells during their autonomous and symplastic growth (summarized in Table 1). Note that the values of the mechanical parameters of the model result in a quasi-equilibrium cell growth regime of the isolated cell when the values of turgor and osmotic pressures are practically the same and constant (≈ 3.6 bar) over the cell cycle (see Figure 2). We performed numerical experiments to investigate the sensitivity of the visible length of an isolated growing cell just before its division. The sensitivity was studied in terms of the variation of the mechanical parameters as described in Section 2.2.1 of the Methods Section. The results demonstrated that the sensitivity of this final cell length to all mechanical parameters decreases with increasing absolute values of the last. The largest sensitivity of the cell visible length was with respect to variation of the coefficient of cell wall growth rate, η. The smallest sensitivity was observed with respect to the variation of the coefficient for turgor pressure, β (see Table 1).

**Figure 3:**
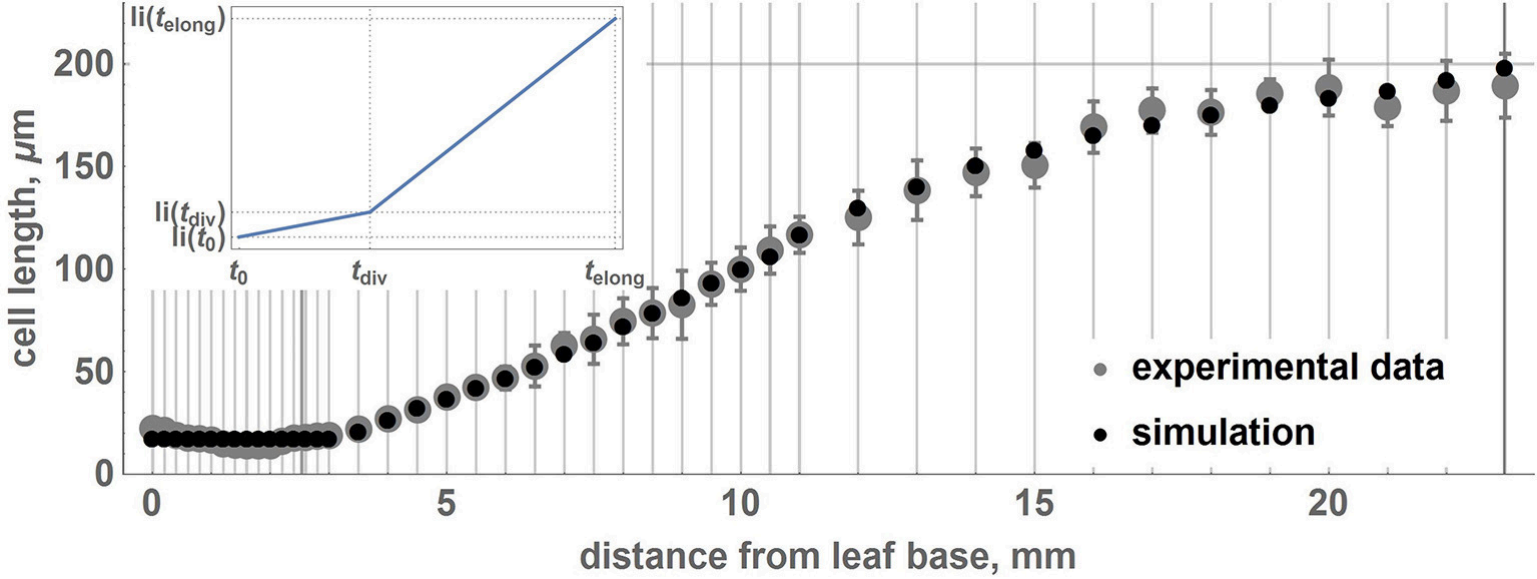
**Relationship between average lengths of cells and distance from the leaf base: comparison between experimental data and the model**. Points correspond to the average lengths of the cells on intervals designated by gray vertical lines. Gray points denote experimental data, and black points denote the results of the simulation with the obtained optimal parameters. The error bars denote the standard errors of the mean. The experimental points and standard errors were obtained by manual digitizing of data on leaf 1 from Beemster et al. (1996). The inset shows a piecewise linear growth function for the isosmotic length of the cell.

The parameters of the piecewise linear cell biomass growth function, *a*_1_ and *a*_2_ (see Equation 15), were estimated by the cell size fitting to the real data, as described in the Section 2. The estimates correspond to the minimum value of the cost function equal to 10^−3^. Figure 3 demonstrates that the two distributions coincide well, suggesting the applicability of our model to reproduce the geometric characteristics of the wheat leaf cells in the growth zone.

Stochastic cell division in the meristematic zone and cell growth mode switching affect the average cell size and its variations over the intervals located at different distances from the leaf base. We performed 100 simulations of wheat leaf growth to estimate the influence of the fluctuation resulting from stochastic nature of the cell division *d* parameter on the cell length distribution along the leaf axis. Each model leaf consisted of 100 cell files with the length of the growth zone equal to that of the real leaf (Table 2). In this way, we generated 100 distributions of the cell length along the model leaf axis. We estimated the mean and the standard deviation of the cell length for each interval from the leaf base using these data. The distribution of the cell length along the axis and their standard deviations in the simulation replicates and real wheat leaf are shown in Figure 3. The figure demonstrates that the two profiles coincide well and that the model is robust with respect to stochastic fluctuations in cell division. These results suggest the applicability of our model to reproduce the geometric characteristics of the wheat leaf cells in the growth zone.

### 3.2. Mechanical behavior of cells during symplastic growth

#### 3.2.1. Increased values of cells osmotic and turgor pressures are characteristics of transition zone

**Figure 4:**
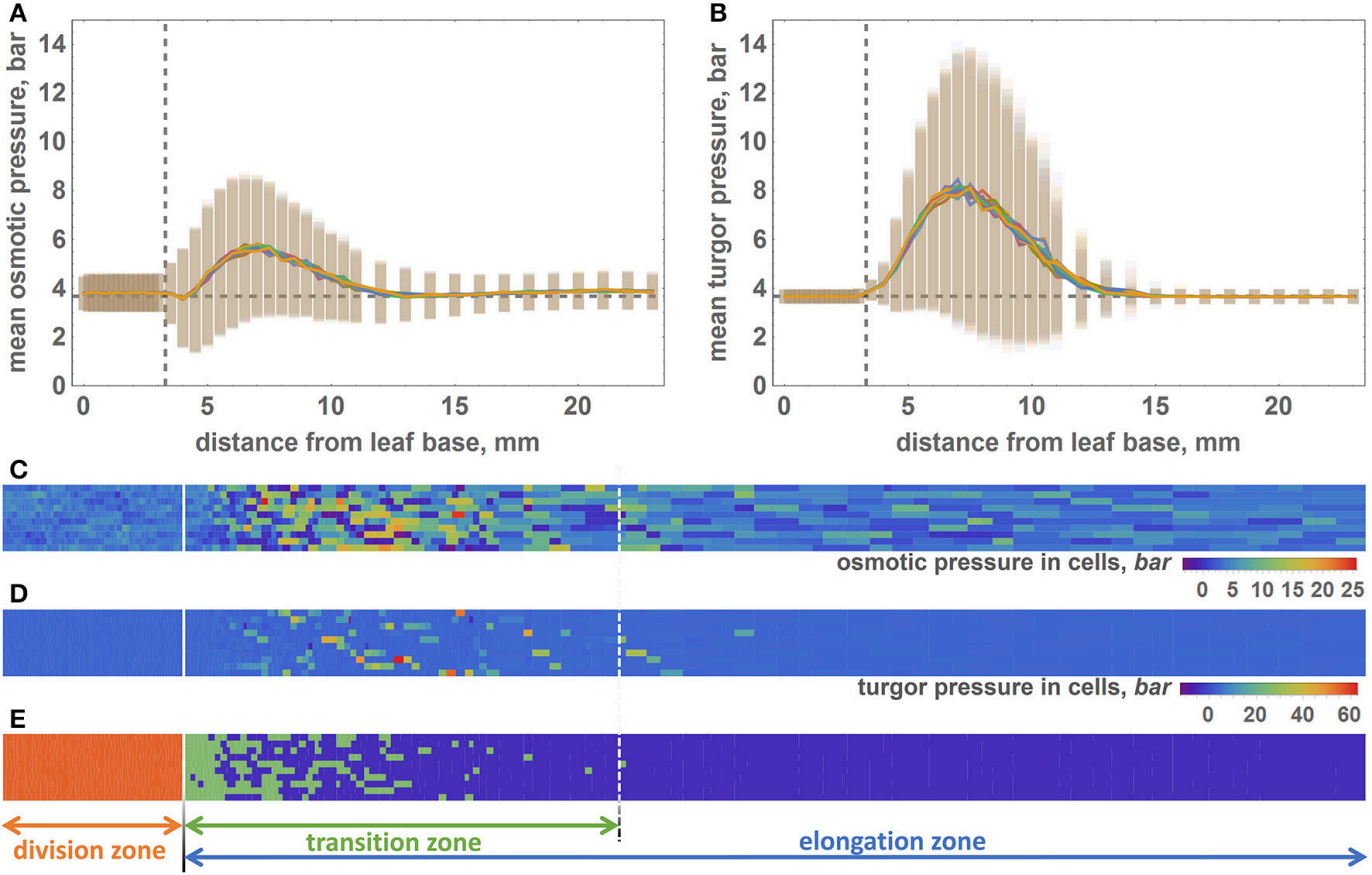
**The distribution of the osmotic and turgor pressures in cells along the model leaf axis. (A)** Mean values (lines) and standard deviations (vertical bars) of the osmotic pressure (Y axis) in cells with respect to their distance from the leaf base (X axis). Vertical dashed line shows the border between the division and transition zones. Horizontal dashed line shows the equilibrium value of the osmotic pressure. **(B)** The mean values (lines) and standard deviations (vertical bars) of the turgor pressure (Y axis) in cells with respect to their distance from the leaf base (X axis). Graph notations are the same as in **(A)**. **(C)** 2D diagram of the osmotic pressure values for the epidermal cells at the time when the simulation stops. Cells represented by the rectangles, whose sizes are proportional to the cell sizes. The osmotic pressure of the cell shown by rectangle color. The color key is shown at the right bottom part of the diagram. Solid white line is the border between the DZ and TZ regions. Dashed white line is the distal border of the TZ. **(D)** 2D diagram of the turgor pressure values for the epidermal cells at the time when the simulation stops. The description of the diagram is the same as for **(C)**. **(E)** Location of cells of different types in the model leaf epidermis. The cells of different types colored by different colors: red for the DZ cells, green for the TZ cells, and violet for the EZ cells. The division of the leaf into three zones is shown by solid and dashed white lines on the diagram and by arrows below the diagram.

In addition to reproducing the geometrical features of the growing leaf, our model is also able to calculate turgor and osmotic pressures for each cell (Equations 7, 8). During the simulation of the leaf growth, we collected information about the turgor and osmotic pressures of the cells and obtained profiles of these pressure averages for the cells at different intervals of distance from the leaf base. To estimate the variability of the pressures within the distance interval, we calculated the standard deviation of the mean for them. The profiles of the osmotic and turgor pressures along the model leaf axis are shown in Figures 4A,B, respectively. These plots demonstrate that in the DZ (distance from the leaf base below 3.3 mm), the average values of the osmotic and turgor pressures are constant with respect to the distance from the leaf base and is ≈4 bar (close to the value obtained for the isolated cell simulation, see Figure 2). At the larger distance from the base, both average pressures increase, reaching a maximum at 7 mm (≈6 bar for osmotic pressure and 8 bar for turgor pressure). With increasing distance from the base above 7 mm, both osmotic and turgor pressures drop to the equilibrated values (≈4 bar) at ≈12.5 and 15 mm from the base for osmotic and turgor pressures, respectively. The pattern of pressure changes corresponds well with the leaf zonation pattern used in our model, namely, constant pressures close to the equilibrium values are observed for DZ and EZ regions; increased pressures are observed for the cells in the TZ.

The pressure variation behavior is similar to the average pressure values. It is constant at distances below 4 mm for both types of pressure. It increases at the interval from 4 to 7 mm and then decreases within the 7–15 mm interval. Interestingly, the absolute values of the turgor pressures are higher, and its variations are lower than those for the osmotic pressures (Figures 4A,B). Some of the cells exhibit extreme values of the turgor pressure, above 40 bar.

The pattern of the pressure values and their variations observed at the profiles (Figures 4A,B) are apparent from the distribution of cell pressure values in the epidermis plane demonstrated in the 2D color diagrams in Figures 4C,D for the osmotic and turgor pressures, respectively. The zonation pattern of the high/low pressure values coincides spatially with the prevalence of the different cell types in the leaf plane (Figure 4E). DZ-type cells are mainly located at distances below 3.3 mm from the base, and TZ cells are located at 4–15 mm from the base. However, note that the location of the distal border of the TZ is not permanent as for the single and for different simulation runs. The majority of the EZ cells are located at distances >15 mm from the base.

#### 3.2.2. Temporal patterns of the cell pressure changes during symplastic growth

**Figure 5:**
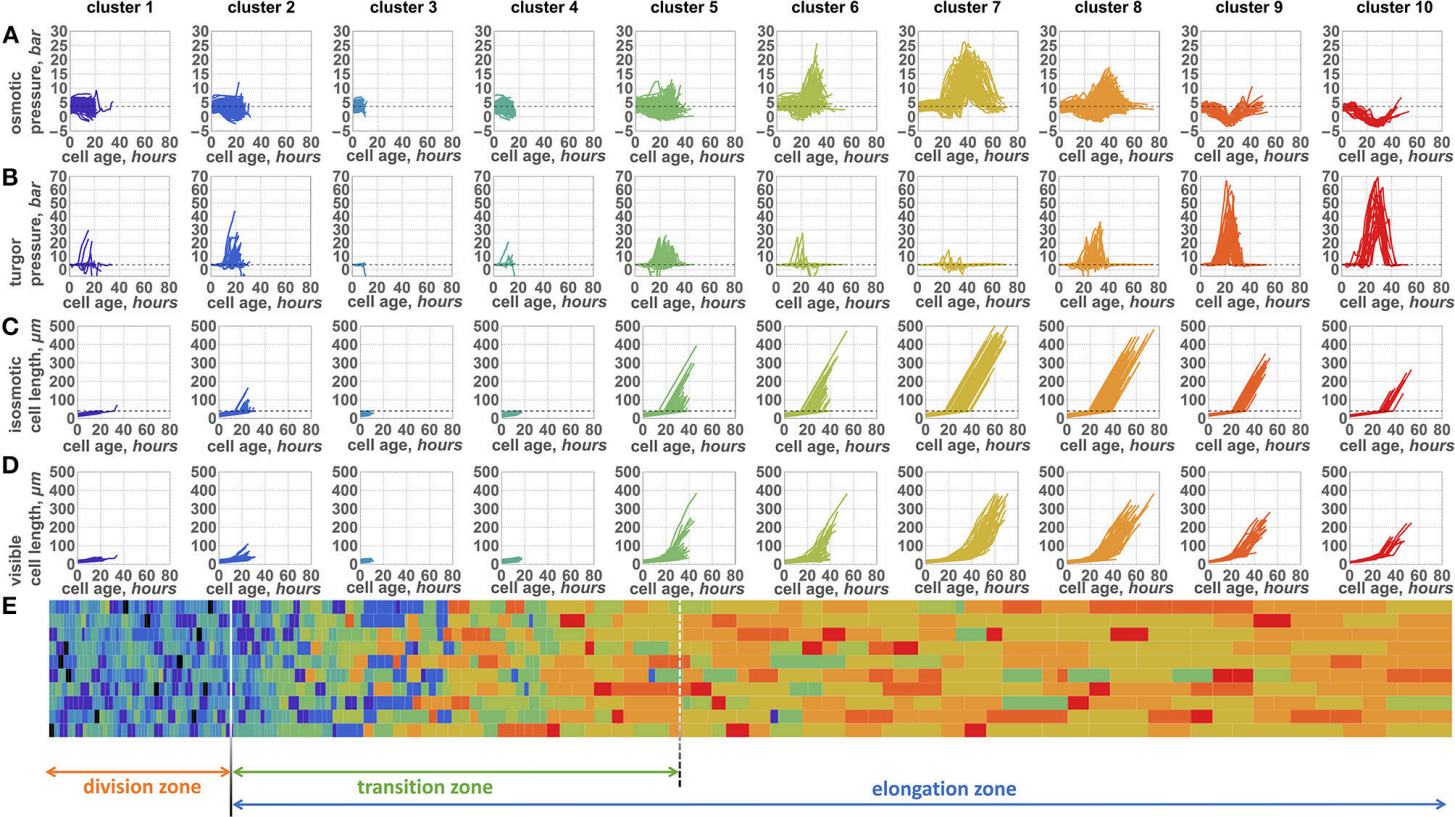
**Ten patterns of changes of internal parameters of the cells during symplastic growth**. Plots of osmotic pressure **(A)**, turgor pressure **(B)**, isosmotic cell length (biomass) **(C)**, and visible length **(D)** (Y axis) with respect to the cell growing time (X axes, hours) are shown. Plot columns represent parameter changes for the cells from the representative clusters 1–10 (cluster numbers shown above the plot columns). Each cluster plot has a specific color. **(E)** 2D diagram of the epidermal cells at the time when the simulation stops. Cells are represented by the rectangles, whose sizes proportional to the cell sizes. The type of the cell (cluster to which cell belongs to) is shown by the same color as the cluster plot (see **A–D**). The division of the leaf into zones is shown by arrows below the diagram.

During the leaf growth simulation, we recorded the osmotic and turgor pressure values of each cell that appeared (≈43,000 cells in 100 cell files in total), as described in Section 2.4 of Methods. We performed cluster analysis of these profiles to reveal groups of cells with similar pressure changes during their symplastic growth. Consequently, we obtained 10 clusters of cells, for which osmotic and turgor pressure, isosmotic cell length (biomass) and visible cell length changes are shown in Figures 5A,D, respectively. We observed separation of all cells into groups with short (clusters 1–4), medium (clusters 5–6), and long (clusters 7–10) lifespans. Cells from the first group exhibit a short time before their division and a constant osmotic pressure during their lifespan. These four clusters differ in turgor pressure/biomass changes. Clusters 1, 3, and 4 exhibit constant values of the turgor pressure for most of the cells. Cells from these clusters mostly reside in the DZ (Figure 5E). Cells from cluster 2 present an increase in both visible size and biomass (Figures 5C,D) at the end of their lifespan. These cells appear in the DZ a short time before the simulation finishes and are likely to enter the TZ soon after their appearance.

The cells from the second group of clusters with a medium lifespan exhibit two distinct types of behavior (Figures 5A–D). One part of them (cluster 5) exhibit relatively constant values of the osmotic pressure and an increase in the turgor pressure during their growth. In contrast, cells from cluster 6 present a significant increase in the osmotic pressure; however, the majority of these cells are characterized by a constant turgor pressure. The size and biomass changes (Figure 5C) for the cells from this group appear to be very similar: they start to increase after a short initial period of constancy. The rate of visible cell length increase is larger for the cluster 5 cells, however (Figure 5D). These cells are mostly located in the TZ with a slight abundance in the EZ zone (Figure 5E) when the simulation stops.

Long-living cells present the largest variability in pressure changes. However, there are several typical profiles represented by clusters 7, 8, 9, and 10. Interestingly, the pressure changes in cells of clusters 7 and 8 are similar to those in clusters 5 and 6, respectively. The characteristics of cluster 7 are a high peak observed for the osmotic pressure with a slight variation in turgor pressure. During its growth time, the cell has passed into the elongation mode; however, some of its neighboring cells have not yet passed this mode. Therefore, the visible length of the cell increased more slowly (Figure 5D) than would be expected for the freely growing cell. In contrast, its isosmotic length (biomass) increases permanently (Figure 5C). This discrepancy between visible cell length and its biomass according to Equation (7) resulted in the sharp increase of the osmotic pressure demonstrated by the peak described above. The cluster 8 cells have a lower osmotic pressure peak but a larger variation of the turgor pressure. The cells from clusters 7 and 8 at the end of the simulation are mostly located in the EZ.

Finally, cells from clusters 9 and 10 exhibit similar behavior. They do not show any significant increase in the osmotic pressure. In contrast, a small decrease in the osmotic pressure is a characteristic of these two clusters: it is shorter in cluster 9 and longer in cluster 10. At the same time, these cells undergo a sharp increase in the turgor pressure, which is the most pronounced among the clusters. The cells from these two clusters are located far from the leaf base when the simulation stops, mostly in the elongation zone. These cells likely appear at the distal part of the division zone. They have a lower biomass growth rate at the time of their appearance (Figure 5C), while the cells from neighboring files entered the fast growth mode. Consequently, the cells from clusters 9 and 10 undergo stretch tension in the TZ, which results in a decrease in the osmotic pressure and an increase in the turgor pressure.

The cluster analysis demonstrates that changes of the cells pressure parameters in the growing symplastic tissues are not uniform within the cell lifespan. There are several typical patterns of the pressure changes, unlike in the independent cell growth (see Figure 2 for comparison). The pattern of the pressure depends specifically (and stochastically) on the cell location, the initial state of the cell parameters, and the behavior of the neighboring cells. Another distinctive feature of the model is the negative relationship between turgor and osmotic pressures (for comparison, see clusters 6–10), which is the intrinsic model property due to Equations (7, 8).

## 4. Discussion

### 4.1. Quasi-one-dimensional model of a unidirectional growth of plant tissue

Experience in the use of vertex-based models for simulating unidirectional growing tissue shows that for the generation of longitudinal rows of cells, which is in particular typical for a linear leaf, an additional constraint on the possible movement of vertices should be introduced. For example, in the model of Vos et al. (2014), vertices are constrained to move only parallel to the longitudinal axis of the root. This allows for the anisotropic elastic-plastic properties of the cell-wall material to be taken into account. In our model, anisotropy in the 2D cell pattern arises due to its quasi-one-dimensional representation (see Section 2.3 of Methods) and does not require additional constrains on the movement of vertices. We model cells rather than cell walls in the formalism of “glued” DL-systems (Zubairova et al., 2015). In this framework, the cells segments are stacked into the cell files and neighboring cell files are glued into the leaf blade. In our model, the boundaries of the stacked fragment are analogs of the vertices of cells in the vertex-based model. We store the information about the correspondence of fragments and cells to the cell fragments as a one-dimensional object (fragmental DL-system). Such a one-dimensional representation can be interpreted such that the absolute shear rigidity of the cell-wall material prevents skew deformation of the cells.

### 4.2. On cell growth function

Note that in the model, we considered the growth of the cell biomass as the only “driving force” for the increase in the osmolytes concentration in the cell. This justifies the introduction of isosmotic cell length, *li*, as a model variable. Therefore, we did not consider the law of biomass conservation in our model. However, considering here the autonomous growth of cells within a tissue, we described this as function of isosmotic length (the equivalent of dry cell biomass in our model) depending on the time.

In the model, we propose a piecewise-linear function of the isosmotic cell length growth. Vos et al. (2014) also considered such a growth function (for the cell target volume) as one of the options in the vertex-based simulation of Arabidopsis root growth. At the same time, in a number of studies (Barlow, 1969; van der Weele et al., 2003) based on experimental data on cell length profiles along the growth axis of the Arabidopsis root, the authors suggested a smooth non-linear growth function for the cell length. Hu and Schmidhalter (2008) also suggested a smooth dependence of the cell length on the time using the data on the distribution of visible lengths of cells along the wheat leaf growth zone. Note that in the above papers, the authors measured the visible cell length, *l*, in terms of our model, and did not consider the difference between “internal” and visible sizes.

**Figure 6:**
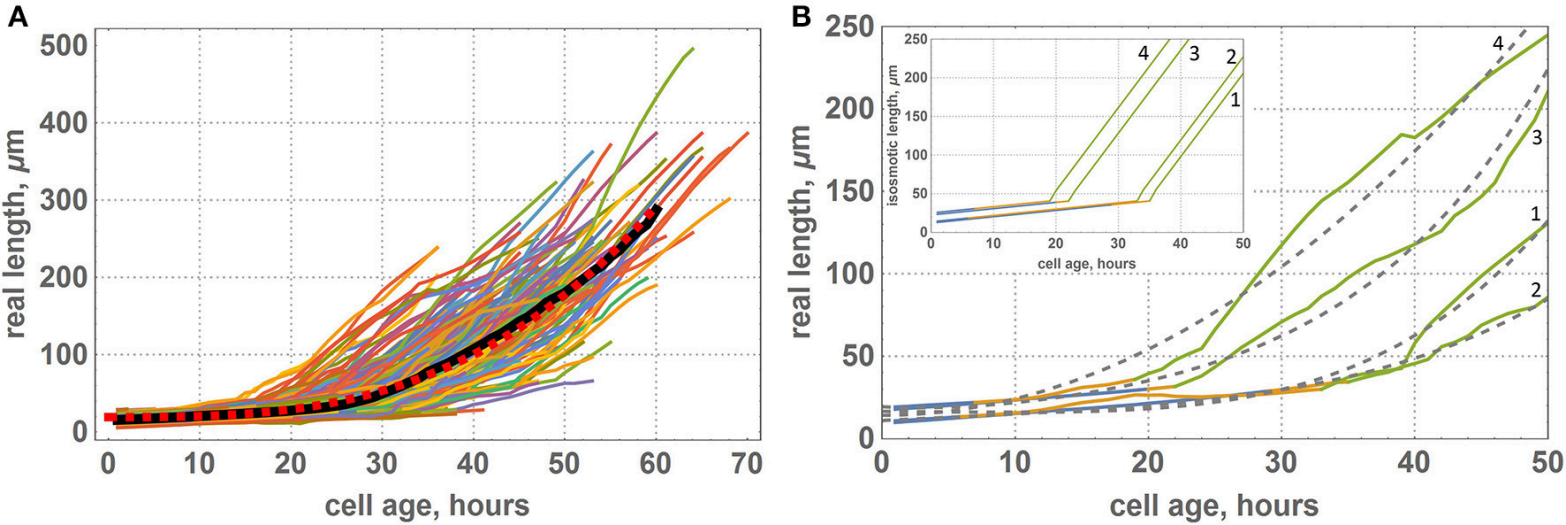
**Growth functions of visible lengths for cells of the “model leaf” within symplastic growth mode. (A)** Growth functions of visible lengths for all cells of the leaf, where the black curve denote the average lengths of cells of the same age and red dashed curve denotes a smooth function approximation [*f*(*x*) = 19.3119 + 0.0013*x*^3^]. **(B)** Growth functions of visible lengths for some selected cells of the leaf: 1st cell had a small initial length and appeared at the proximal part of the DZ, 2nd cell had a small initial length and appeared at the distal part of the DZ, 3rd cell had a large initial length and appeared at the proximal part of the DZ, and 4th cell had a large initial length and appeared at the distal part of DZ. The color of the curve denotes the growth zone: blue is for the DZ, yellow is for transition zone, and green is for the EZ. Gray dashed curves denote a smooth approximation with a polynomial function. The inset shows the corresponding growth functions for isosmotic length.

In our simulations, we set a piecewise-linear growth function of the isosmotic cell length (inset in Figure 6B); however, the results of our computational experiments showed a smooth non-linear function for the visible cell growth functions (Figure 6A). We suppose that smoothing occurs due to the fact that under the terms of symplastic growth, cells expand and contract each other due to the different growth rates of neighboring cells. As shown in Figure 6B, the growth functions and smooth non-linear approximations are different for various cells and depend on the cells neighborhood. Figure 6 shows growth functions for four cells, which had different initial lengths (the 1st and the 2nd cells had a small initial length, and the 3rd and the 4th cells had a larger one) and grew under different mechanical conditions. According to our model, cells underwent the largest mechanical stress from neighbors in the transition zone between the DZ and EZ (marked in yellow on the growth curves in Figure 6B). The distance from the base for the cell at the time of its appearance in the DZ strongly determines the time that this cell will spend in a TZ. For example, the 1st and the 3rd cells (Figure 6B) appeared in the proximal part of the DZ and passed the transition zone relatively quickly. The rate of their growth is greater than the average for the neighboring cells. Consequently, these cells are “compressed” by neighbors. This behavior is typical for the cells from clusters 6 and 7 (Figure 5). The 2nd and the 4th cells (Figure 6B) appeared in the distal part of the DZ and passed the transition zone slowly. Therefore, they grew with a lower rate in comparison with the neighbors. Hence, these cells were “stretched” by them. This behavior is typical for cells from clusters 9 and 10 (Figure 5).

Thus, the problem of interpreting the data on cell growth rate in the tissue requires careful consideration. The growth functions of visible lengths may also vary from cell to cell because the cells in the tissue are in the stress-strain state, which may vary depending on the environment and time. In this view, the cell typical dynamics is the time dependence of the ratio of molecular genetic markers (Sablowski and Dornelas, 2014), dry biomass dynamics, or, as in our model, the isosmotic cell length. Therefore, the question arises of which cell characteristics should be assessed in experimental observations to characterize cell growth regulation in the tissue.

### 4.3. The growth function for relaxed cell length and quasi-equilibrium cell growth mode

The regulation of the synthesis of the cell-wall material is still the subject of study (Carpita and Gibeaut, 1993; Braybrook and Jönsson, 2016; Chen et al., 2016; Wang et al., 2016; Zhang et al., 2016). In this study, we proposed that the growth rate of the relaxed length is proportional to the growth rate of the isosmotic length (we can interpret *dli*/*dt* as the growth rate of dry biomass). The control function (term ${\left(P_{turg}-P_{c}\right)}^{3}$ in Equation 11) determines that the threshold value of turgor pressure is the target control parameter. The 3rd power of the pressure difference in Equation (11) along with the mechanical parameter values (Table 1) result in a rapid growth of the cell wall in our model. Consequently, the autonomous growth of a single cell occurs at a steady turgor pressure. These parameter values provide mechanical equilibrium when the turgor pressure is equal to the osmotic pressure in the case of isolated cells (Figure 2). This mode of cell growth in tissue was chosen because in the framework of vertex-based models (Nagai and Honda, 2001; Dupuy et al., 2008; Merks et al., 2011), and the computational algorithm assumes that equilibrium is achieved at each iteration step. In particular, Vos et al. (2014) used such an algorithm for modeling root growth based on the dynamics of vertices with additional constraints on its movements.

### 4.4. The transition between division and elongation zones

Despite the simplicity of the growth zone structure for unidirectional (linear) growing plant organs (roots, hypocotyl, and leaves of monocots), there are several hypotheses about possible mechanisms for the formation of its structure (Baluska et al., 1996; Verbelen et al., 2006; Benková and Hejátko, 2009; Baluška et al., 2010; Cederholm et al., 2012; Ivanov and Dubrovsky, 2013; Avramova et al., 2015). The influence of the auxin concentrations on the relative growth rate in different growth zones of the root was investigated in Chavarría-Krauser et al. (2005). The authors constructed a 1D biophysical model of auxin-related control for a single cell file. Some mechanisms of cell transition between the DZ and EZ were tested for the root growth model in Vos et al. (2014). The authors of this paper concluded that the formation of the zone size is influenced by phytohormone concentrations.

In experimental work on the maize leaf (Nelissen et al., 2012), the authors also showed that the concentrations of phytohormones may define a transition between division and elongation zones. In our model, we also assumed that gradients of morphogen substance execute patterning of the leaf growth zone in a concentration-dependent manner and accepted a threshold mechanism of cell transition from a state of proliferation to accelerated growth while the cell size may increase tenfold. The growth and division of cells located before the cell in the same longitudinal row and the cell's own growth result in movement of the cell in the direction from the leaf base to the tip. When a cell is shifted at a certain distance from the leaf base, the concentration of morphogen substance decreases and the cell loses its ability to divide and enters the so-called transition zone, where it continues to grow with the same (slow) rate until its initial size reaches the threshold length. Subsequently, the cell turns itself into a state of expansion growth, when it begins a rapid increase in size that is likely due to the combination of endoreduplication and vacuolization (Perrot-Rechenmann, 2010; Barrada et al., 2015). Since all cells pass this switching phase asynchronously, a wide area of the leaf formed where some cells switched to fast growth and some cells continued to grow with at a slow rate (as meristematic cells). Such a neighborhood defines the transition zone (Ivanov and Dubrovsky, 2013). Furthermore, within the same cell file, the distance from the leaf base where the cell switches to another phase of growth may be different (Lück et al., 1997).

In our model, we assumed that there exists a division zone boundary defined by a concentration gradient of a morphogen. Beyond that zone, the cell continues to grow at the same slow rate as in the division zone until reaching a critical size. Then, the cell growth mode changes. Thus, the growth zone of the leaf has the following structure: the meristematic zone is in the leaf base where cells can divide, a transition zone, and then the elongation zone. This structure of leaf growth area is consistent with the structure of the growth zones for Arabidopsis roots and maize leaves considered in Avramova et al. (2015).

### 4.5. The mechanical behavior of cells during the symplastic growth of the linear leaf

The computational experiments considered in this paper, as well as in Vos et al. (2014), showed that it is possible to choose model parameters to reproduce the experimentally observed geometric pattern of cell tissue structure. However, the mechanical parameters of the cells during symplastic growth remain hidden because vertex-based models (Nagai and Honda, 2001; Dupuy et al., 2008; Merks et al., 2011) use abstract potential forces for simulating changes in the geometry of cells. Consequently, these potentials did not allow directly interpreting the mechanics of plant cell growth. Here, we reduced the dimension and switched to the quasi-one-dimensional model to describe the 2D leaf growth. This allowed us to explicitly link the changes in cell volume and the deformation of the cell wall. Consequently, we observed the dynamics of osmotic and turgor pressures in cells during their growth.

The significant “jumps” of pressure in the cells could be observed in the real nature. For instance, the original experimental data from Dyson et al. (2014) can serve as an indirect confirmation of such significant changes of pressure in the cells. Although, the authors conclude that the effective (average) turgor pressure does not change throughout the different growth zones along the Arabidopsis root (Figure 3A from Dyson et al., 2014), their measurements indicate that pressure in individual cells of the root along the growth axis changed from 1 to 4 atm., i.e., 4 times. Note that the pressure measurements in Dyson et al. (2014) were performed in single cells for a short time only.

In our model, cells grow autonomously, but as a part of symplastic growing tissue. Note that in the framework of vertex-based models of plant tissue, the autonomous cell growth is usually accepted (Nagai and Honda, 2001; Dupuy et al., 2008). As we suppose, this could be one of the possible reasons why extreme values of pressure are observed for the cells in the transition zone between the DZ and EZ (up to 50 bar, see Figures 5A,B). Our computational experiments demonstrated that the autonomous growth of cell biomass may cause significant deformation of the cell-wall due to cell stretching/compression by the neighboring cells growing at different rates. From the other hand, extreme values of pressure are also likely to arise from the assumption of the absolute shear rigidity of the cell-wall material.

The model framework suggested here allows for the non-autonomous cell growth to be taken into account. The first step is to determine the necessity and the way the cell regulates its growth. The second step is the determination of the coordination of cells of the tissue as a whole during its growth.

## 5. Conclusion

A model of the mechanics of the symplastic growth of a linear leaf is proposed. The model is a special case of a class of vertex-based models, and its parameters can be meaningfully interpreted. The biomechanical description of the autonomous growth of individual plant cells was based on explicit expressions of turgor and osmotic pressures as functions of the cell lengths. The model was developed in the framework of Lockhart (1965) and Ortega (2010) approaches. To illustrate the consequences of a widely used (in the framework of the vertex-based approach) model assumption of the autonomous growth of cells in the tissue, we used an explicit function depending only on the time for cell biomass growth.

Consideration of the geometry of the unidirectional growth of a linear leaf allowed us to bind the cell volume change with the change of the cells walls and to describe osmotic and turgor pressures in the following state variables of growing cells visible length, *l*, isosmotic length, *li*, and relaxed length, *lr*. The model of cell growth in a plant tissue was constructed from consideration of the mechanical forces arising between cells in the symplastic growing tissue. It was shown that the proposed model of cell growth as a part of symplastic growing tissue subject to certain parameter values provides a good approximation of the experimental data on the growth of wheat leaf. At the same time, the physical interpretability of the model variables was able to reveal significant variations of turgor and osmotic pressures in cells of growing tissue. The question of accordance of such dynamics of pressures to the real situation requires experimental verification, and it is critically important for judging the adequacy of the model, particularly the assumptions about the autonomous growth of cells and the rule of cell transition between the DZ and EZ.

To conclude, we want to emphasize the importance of cell-based biomechanical models of the plant tissue morphodynamics allowing the estimation of mechanical stress in cell walls because they can serve as regulatory signals for molecular genetic systems underlying the mechanism of the individual cell growth and formation of biomechanical properties of the cell. Such models make it possible to link biomechanical and molecular genetic levels of morphogenesis description and allow us to make a step toward a complex integrated model of morphogenesis.

## Author contributions

UZ developed the model and its computational implementation, conducted the computational experiments, wrote the manuscript, and drew the figures. SN designed the research, developed the model and its computational implementation, and wrote the manuscript. AP designed the program, ran the calculations, and provided instructive comments on the original manuscript. NP strengthened the introduction and discussion from a biological perspective and commented on the whole manuscript. SG made substantial contributions to the model of cell biomechanics. DA and NK conceived the topic and problem and provided instructive comments on the original manuscript.

## Funding

The development of the model for the symplastic growth of a linear leaf accounting for the structure of the growth zone and approximation of experimental cell length profiles was supported by the Russian Science Foundation according to research project No. 14-14-00734. Simulations were performed using a program developed with support of the ICG SB RAS budget project No. 0324-2015-0003.

### Conflict of interest statement

The authors declare that the research was conducted in the absence of any commercial or financial relationships that could be construed as a potential conflict of interest.

## References

[B1] Ali O. Traas J. (2016). Force-driven polymerization and turgor-induced wall expansion. Trends Plant Sci. 21, 398–409. 10.1016/j.tplants.2016.01.019 26895732

[B2] Avramova V. Sprangers K. Beemster G. T. (2015). The maize leaf: another perspective on growth regulation. Trends Plant Sci. 20, 787–797. 10.1016/j.tplants.2015.09.002 26490722

[B3] Baluška F. Mancuso S. Volkmann D. Barlow P. W. (2010). Root apex transition zone: a signalling–response nexus in the root. Trends Plant Sci. 15, 402–408. 10.1016/j.tplants.2010.04.007 20621671

[B4] Baluska F. Volkmann D. Barlow P. W. (1996). Specialized zones of development in roots: view from the cellular level. Plant Physiol. 112:3. 10.1104/pp.112.1.3 11536754 PMC157916

[B5] Barlow P. (1969). Cell growth in the absence of division in a root meristem. Planta 88, 215–223. 24504892 10.1007/BF00385064

[B6] Barrada A. Montané M.-H. Robaglia C. Menand B. (2015). Spatial regulation of root growth: placing the plant tor pathway in a developmental perspective. Int. J. Mol. Sci. 16, 19671–19697. 10.3390/ijms160819671 26295391 PMC4581319

[B7] Bayer E. M. Smith R. S. Mandel T. Nakayama N. Sauer M. Prusinkiewicz P.. (2009). Integration of transport-based models for phyllotaxis and midvein formation. Genes Dev. 23, 373-384. 10.1101/gad.497009 19204121 PMC2648550

[B8] Beemster G. T. Masle J. Williamson R. E. Farquhar G. D. (1996). Effects of soil resistance to root penetration on leaf expansion in wheat (*Triticum aestivum* L.): kinematic analysis of leaf elongation. J. Exp. Bot. 47, 1663–1678.

[B9] Benková E. Hejátko J. (2009). Hormone interactions at the root apical meristem. Plant Mol. Biol. 69, 383–396. 10.1007/s11103-008-9393-6 18807199

[B10] Braybrook S. A. Jönsson H. (2016). Shifting foundations: the mechanical cell wall and development. Curr. Opin. Plant Biol. 29, 115–120. 10.1016/j.pbi.2015.12.009 26799133

[B11] Carpita N. C. Gibeaut D. M. (1993). Structural models of primary cell walls in flowering plants: consistency of molecular structure with the physical properties of the walls during growth. Plant J. 3, 1–30. 8401598 10.1111/j.1365-313x.1993.tb00007.x

[B12] Cederholm H. M. Iyer-Pascuzzi A. S. Benfey P. N. (2012). Patterning the primary root in *arabidopsis*. Wiley Interdiscip. Rev. 1, 675–691. 10.1002/wdev.49 23799568

[B13] Chaiwanon J. Wang W. Zhu J.-Y. Oh E. Wang Z.-Y. (2016). Information integration and communication in plant growth regulation. Cell 164, 1257–1268. 10.1016/j.cell.2016.01.044 26967291 PMC5126258

[B14] Chavarría-Krauser A. Jäger W. Schurr U. (2005). Primary root growth: a biophysical model of auxin-related control. Funct. Plant Biol. 32, 849–862. 10.1071/FP05033 32689182

[B15] Chen S. Jia H. Zhao H. Liu D. Liu Y. Liu B.. (2016). Anisotropic cell expansion is affected through the bidirectional mobility of cellulose synthase complexes and phosphorylation at two critical residues on CESA3. Plant Physiol. 171, 242–250. 10.1104/pp.15.01874 26969722 PMC4854686

[B16] Chickarmane V. S. Gordona S. P. Tarra P. T. Heisler M. G. Meyerowitz E. M. (2012). Cytokinin signaling as a positional cue for patterning the apical-basal axis of the growing *arabidopsis* shoot meristem. Proc. Natl. Acad. Sci. U.S.A. 109, 4002–4007. 10.1073/pnas.1200636109 22345559 PMC3309735

[B17] Cosgrove D. J. (2005). Growth of the plant cell wall. Nat. Rev. Mol. Cell Biol. 6, 850–861. 10.1038/nrm1746 16261190

[B18] Dupuy L. MacKenzie J. Rudge T. Haseloff J. (2008). A system for modelling cell-cell interactions during plant morphogenesis. Ann. Bot. 101, 1255–1265. 10.1093/aob/mcm235 17921524 PMC2710276

[B19] Dyson R. J. Band L. R. Jensen O. E. (2012). A model of crosslink kinetics in the expanding plant cell wall: yield stress and enzyme action. J. Theor. Biol. 307, 125–136. 10.1016/j.jtbi.2012.04.035 22584249 PMC3414840

[B20] Dyson R. J. Vizcay-Barrena G. Band L. R. Fernandes A. N. French A. P. Fozard J. A.. (2014). Mechanical modelling quantifies the functional importance of outer tissue layers during root elongation and bending. New Phytol. 202, 1212–1222. 10.1111/nph.12764 24641449 PMC4286105

[B21] Gibson L. J. (2012). The hierarchical structure and mechanics of plant materials. J. R. Soc. Interface 9, 2749–2766. 10.1098/rsif.2012.0341 22874093 PMC3479918

[B22] Glazier J. A. Graner F. (1993). Simulation of the differential adhesion driven rearrangement of biological cells. Phys. Rev. E 47, 2128. 9960234 10.1103/physreve.47.2128

[B23] Graner F. Glazier J. A. (1992). Simulation of biological cell sorting using a two-dimensional extended potts model. Phys. Rev. Lett. 69, 2013. 10046374 10.1103/PhysRevLett.69.2013

[B24] Green P. B. (1999). Expression of pattern in plants: combining molecular and calculus-based biophysical paradigms. Am. J. Bot. 86, 1059–1076. 10449383

[B25] Hamant O. Heisler M. G. Jönsson H. Krupinski P. Uyttewaal M. Bokov P.. (2008). Developmental patterning by mechanical signals in *arabidopsis*. Science 322, 1650–1655. 10.1126/science.1165594 19074340

[B26] Hamant O. Traas J. (2010). The mechanics behind plant development. New Phytol. 185, 369–385. 10.1111/j.1469-8137.2009.03100.x 20002316

[B27] Hu Y. Schmidhalter U. (2008). Spatial and temporal quantitative analysis of cell division and elongation rate in growing wheat leaves under saline conditions. J. Integr. Plant Biol. 50, 76–83. 10.1111/j.1744-7909.2007.00379.x 18666954

[B28] Hukin D. Doering-Saad C. Thomas C. R. Pritchard J. (2002). Sensitivity of cell hydraulic conductivity to mercury is coincident with symplasmic isolation and expression of plasmalemma aquaporin genes in growing maize roots. Planta 215, 1047–1056. 10.1007/s00425-002-0841-2 12355166

[B29] Ivanov V. B. Dubrovsky J. G. (2013). Longitudinal zonation pattern in plant roots: conflicts and solutions. Trends Plant Sci. 18, 237–243. 10.1016/j.tplants.2012.10.002 23123304

[B30] Jönsson H. Heisler M. G. Shapiro B. E. Meyerowitz E. M. Mjolsness E. (2006). An auxin-driven polarized transport model for phyllotaxis. Proc. Natl. Acad. Sci. U.S.A. 103, 1633–1638. 10.1073/pnas.0509839103 16415160 PMC1326488

[B31] Kalve S. De Vos D. Beemster G. T. (2014). Leaf development: a cellular perspective. Front. Plant Sci. 5:362. 10.3389/fpls.2014.00362 25132838 PMC4116805

[B32] Krupinski P. Bozorg B. Larsson A. Pietra S. Grebe M. Jönsson H. (2016). A model analysis of mechanisms for radial microtubular patterns at root hair initiation sites. Front. Plant Sci. 7:1560. 10.3389/fpls.2016.01560 27840629 PMC5083785

[B33] Lekka M. Pogoda K. Gostek J. Klymenko O. Prauzner-Bechcicki S. Wiltowska-Zuber J.. (2012). Cancer cell recognition–mechanical phenotype. Micron 43, 1259–1266. 10.1016/j.micron.2012.01.019 22436422

[B34] Lindenmayer A. (1968). Mathematical models for cellular interaction in development, parts i and ii. J. Theor. Biol. 18, 280–315. 5659071 10.1016/0022-5193(68)90079-9

[B35] Lockhart J. A. (1965). An analysis of irreversible plant cell elongation. J. Theor. Biol. 8, 264–275. 5876240 10.1016/0022-5193(65)90077-9

[B36] Lück J. Barlow P. Lück H. (1997). An automata-theoretical model of meristem development as applied to the primary root of *Zea mays* L. Ann. Bot. 79, 375–389.

[B37] Lv H. Li L. Sun M. Zhang Y. Chen L. Rong Y.. (2015). Mechanism of regulation of stem cell differentiation by matrix stiffness. Stem Cell Res. Ther. 6:1. 10.1186/s13287-015-0083-4 26012510 PMC4445995

[B38] Merks R. M. Guravage M. Inzé D. Beemster G. T. (2011). Virtualleaf: an open-source framework for cell-based modeling of plant tissue growth and development. Plant Physiol. 155, 656–666. 10.1104/pp.110.167619 21148415 PMC3032457

[B39] Milani P. Braybrook S. A. Boudaoud A. (2013). Shrinking the hammer: micromechanical approaches to morphogenesis. J. Exp. Bot. 64, 4651–4662. 10.1093/jxb/ert169 23873995

[B40] Milani P. Gholamirad M. Traas J. Arnéodo A. Boudaoud A. Argoul F.. (2011). *In vivo* analysis of local wall stiffness at the shoot apical meristem in *arabidopsis* using atomic force microscopy. Plant J. 67, 1116–1123. 10.1111/j.1365-313X.2011.04649.x 21605208

[B41] Mishra S. R. (2004). Translocation in Plants. New Delhi: Discovery Publishing House.

[B42] Nagai T. Honda H. (2001). A dynamic cell model for the formation of epithelial tissues. Philos. Mag. B 81, 699–719. 10.1080/13642810108205772

[B43] Nelissen H. Rymen B. Jikumaru Y. Demuynck K. Van Lijsebettens M. Kamiya Y.. (2012). A local maximum in gibberellin levels regulates maize leaf growth by spatial control of cell division. Curr. Biol. 22, 1183–1187. 10.1016/j.cub.2012.04.065 22683264

[B44] Nikolaev S. V. Zubairova U. S. Fadeev S. I. Mjolsness E. Kolchanov N. A. (2011). Study of a one-dimensional model, accounting for cell division, of regulation of the renewing zone size in a biological tissue. J. Appl. Ind. Math. 4, 601–611. 10.1134/S1990478911040156

[B45] Nobel P. (2009). Physicochemical and Environmental Plant Physiology. Oxford: Academic Press.

[B46] Noble D. (2008). The Music of Life: Biology Beyond Genes. Oxford: Oxford University Press.

[B47] Ortega J. K. (2010). Plant cell growth in tissue. Plant Physiol. 154, 1244–1253. 10.1104/pp.110.162644 20739609 PMC2971603

[B48] Perrot-Rechenmann C. (2010). Cellular responses to auxin: division versus expansion. Cold Spring Harb. Perspect. Biol. 2:a001446. 10.1101/cshperspect.a001446 20452959 PMC2857164

[B49] Prusinkiewicz P. Hammel M. Mjolsness E. (1993). Animation of plant development, in Proceedings of SIGGRAPH 93, In Computer Graphics Proceedings, Annual Conference Series (Anaheim, CA: ACM SIGRAPH), 351–360.

[B50] Robinson S. Burian A. Couturier E. Landrein B. Louveaux M. Neumann E. D.. (2013). Mechanical control of morphogenesis at the shoot apex. J. Exp. Bot. 64, 4729–4744. 10.1093/jxb/ert199 23926314

[B51] Routier-Kierzkowska A.-L. Smith R. S. (2013). Measuring the mechanics of morphogenesis. Curr. Opin. Plant Biol. 16, 25–32. 10.1016/j.pbi.2012.11.002 23218971

[B52] Sablowski R. Dornelas M. C. (2014). Interplay between cell growth and cell cycle in plants. J. Exp. Bot. 65, 2703–2714. 10.1093/jxb/ert354 24218325

[B53] Sassi M. Traas J. (2015). When biochemistry meets mechanics: a systems view of growth control in plants. Curr. Opin. Plant Biol. 28, 137–143. 10.1016/j.pbi.2015.10.005 26583832

[B54] Shapiro B. E. Meyerowitz E. M. Mjolsness E. (2013). Using cellzilla for plant growth simulations at the cellular level. Front. Plant Sci. 4:408. 10.3389/fpls.2013.00408 24137172 PMC3797531

[B55] Streichan S. J. Hoerner C. R. Schneidt T. Holzer D. Hufnagel L. (2014). Spatial constraints control cell proliferation in tissues. Proc. Natl. Acad. Sci. U.S.A. 111, 5586–5591. 10.1073/pnas.1323016111 24706777 PMC3992650

[B56] Sugimura K. Lenne P.-F. Graner F. (2016). Measuring forces and stresses *in situ* in living tissues. Development 143, 186–196. 10.1242/dev.119776 26786209

[B57] Swarup R. Kramer E. M. Perry P. Knox K. Leyser H. M. Haseloff J.. (2005). Root gravitropism requires lateral root cap and epidermal cells for transport and response to a mobile auxin signal. Nat. Cell Biol. 7, 1057–1065. 10.1038/ncb1316 16244669

[B58] Tardieu F. Reymond M. Hamard P. Granier C. Muller B. (2000). Spatial distributions of expansion rate, cell division rate and cell size in maize leaves: a synthesis of the effects of soil water status, evaporative demand and temperature. J. Exp. Bot. 51, 1505–1514. 10.1093/jexbot/51.350.1505 11006302

[B59] van der Weele C. M. Jiang H. S. Palaniappan K. K. Ivanov V. B. Palaniappan K. Baskin T. I. (2003). A new algorithm for computational image analysis of deformable motion at high spatial and temporal resolution applied to root growth. Roughly uniform elongation in the meristem and also, after an abrupt acceleration, in the elongation zone. Plant Physiol. 132, 1138–1148. 10.1104/pp.103.021345 12857796 PMC526269

[B60] Verbelen J.-P. De Cnodder T. Le J. Vissenberg K. Baluška F. (2006). The root apex of *Arabidopsis thaliana* consists of four distinct zones of growth activities: meristematic zone, transition zone, fast elongation zone and growth terminating zone. Plant Signal. Behav. 1, 296–304. 10.4161/psb.1.6.3511 19517000 PMC2634244

[B61] Vos D. D. Vissenberg K. Broeckhove J. Beemster G. T. S. (2014). Putting theory to the test: which regulatory mechanisms can drive realistic growth of a root? PLoS Comput. Biol. 10:e1003910. 10.1371/journal.pcbi.1003910 25358093 PMC4214622

[B62] Wang T. McFarlane H. E. Persson S. (2016). The impact of abiotic factors on cellulose synthesis. J. Exp. Bot. 67, 543–552. 10.1093/jxb/erv488 26552883

[B63] Wells R. G. (2008). The role of matrix stiffness in regulating cell behavior. Hepatology 47, 1394–1400. 10.1002/hep.22193 18307210

[B64] Zhang Y. Nikolovski N. Sorieul M. Vellosillo T. McFarlane H. E. Dupree R.. (2016). Golgi-localized stello proteins regulate the assembly and trafficking of cellulose synthase complexes in *arabidopsis*. Nat. Commun. 7:11656. 10.1038/ncomms11656 27277162 PMC4906169

[B65] Zubairova U. Golushko S. Penenko A. Nikolaev S. (2015). A computational model of the effect of symplastic growth on cell mechanics in a linear leaf blade. J. Bioinform. Comput. Biol. 13:1540005. 10.1142/S0219720015400053 25556842

